# Non-surgical Endodontic Management of Multiple Mishaps: A Case Report

**DOI:** 10.7759/cureus.59162

**Published:** 2024-04-27

**Authors:** Faisal Alnassar

**Affiliations:** 1 Department of Restorative and Prosthetic Dental Sciences, College of Dentistry, Majmaah University, Al-Majmaah, SAU

**Keywords:** extruted gutta-percha, endodontic mishaps, missed canal, separated file, root canal anatomy

## Abstract

The description of non-surgical retreatment is to eliminate all previous filling materials and correct any mishaps. An adequate understanding of root and canal morphology is a fundamental requirement for obtaining a favorable outcome in endodontic treatment. The endodontic diagnosis was previously treated through therapy with symptomatic apical periodontitis. Treatment was performed under a dental operative microscope. Gates Glidden (GG) drills size 2 and 3 were used to remove the coronal part of gutta-percha for all canals. A braiding technique with a Hedstrom file size 15 without a solvent was used to remove gutta-percha and separate the file together for the palatal canal whereas the mesiobuccal canal was retreated by ProTaper Retreatment Kit. Proper understanding of root canal morphology and using an endodontic armamentarium could reduce endodontic mishaps. This case report described the successful management of overextended gutta-percha, a separated file, and a missed canal.

## Introduction

The main goal of endodontic retreatment is to effectively clean the root canals by removing irritants, particularly microbes that have persisted or invaded the canals following previous treatment. Retreatments, which involve repeating the stages of root canal therapy, should be performed whenever feasible, with biological justification [1]. Following a thorough cleaning and shaping process, the proper sealing of the root canal system is of utmost importance for achieving endodontic success [2]. Ideally, all materials used to fill root canals should be confined to the root canal system. Nevertheless, a complication that often arises during obturation is overextension, particularly in cases involving immature, resorbed, or overinstrumented root canal apices [3]. Microorganisms are the primary causes of treatment failure in root canal systems. Missed canals, ledge formation, fractured instruments, and anatomical complexities, such as isthmuses, and apical ramifications, can lead to intraradicular infections [4]. Accordingly, intraradicular biofilm removal is the primary objective of root canal retreatments and is essential to ensure long-term success [5]. Post-treatment disease may arise due to missed canals and inadequate cleaning of the root canal space, particularly inadequate chemical and mechanical cleaning during biomechanical instrumentation. The presence of a preoperative periapical lesion, apical extent of root filling, and quality of coronal restoration are crucial factors affecting the outcomes of endodontic retreatment [6]. Complete removal of the filling material during orthograde endodontic retreatment is imperative to facilitate thorough cleaning, shaping, disinfection, and subsequent filling of the root canal system. Instrument fracture is an undesirable and troublesome incident during RCT that frustrates both practitioners and patients. It can happen even to experienced clinicians following the most appropriate preventive measures. Complications can occur during many endodontic procedures. However, the best way to deal with endodontic mishaps is prevention. This case report shows a root canal failure with multiple endodontic mishaps and successful management.

## Case presentation

**Figure 1:**
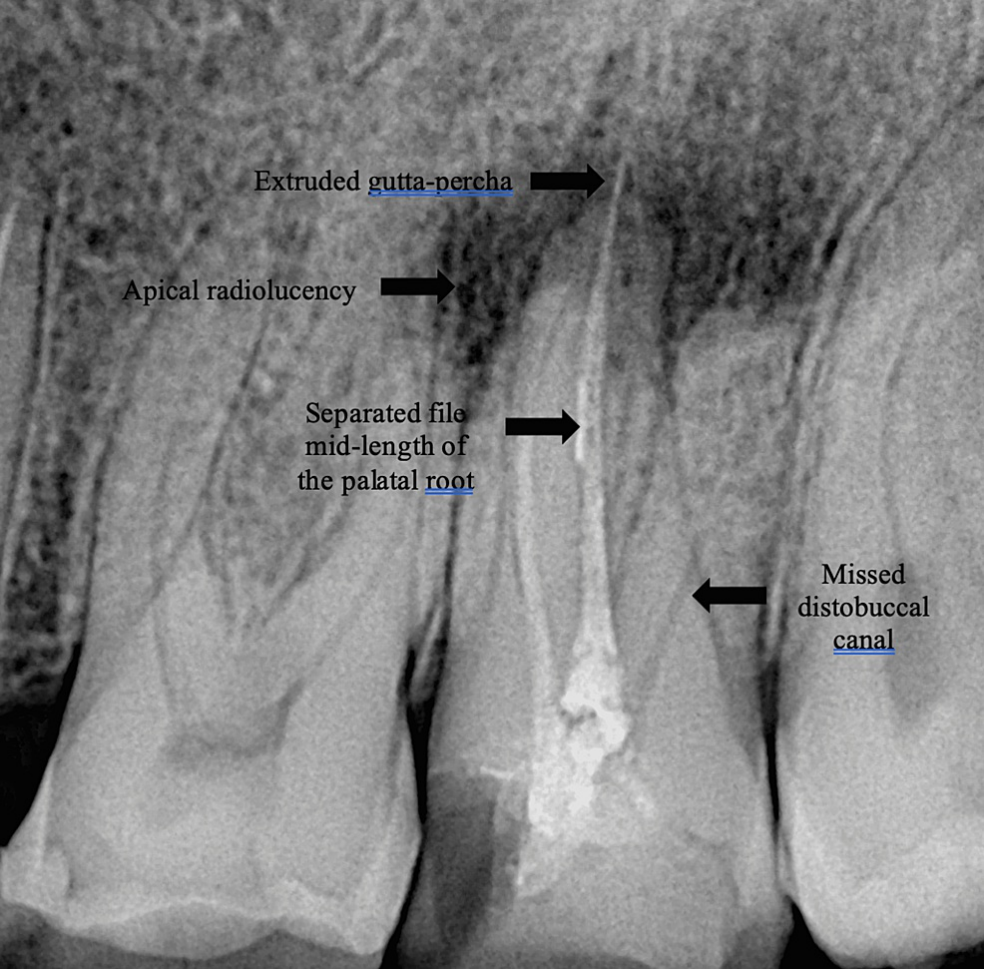
Preoperative radiograph of a maxillary left second molar with dental caries, substantial coronal destruction, missed canal, separated file, extruded gutta-percha, and periapical radiolucency.

A 35-year-old male patient complained of pain while chewing food for one month. The patient's medical history was inconclusive. The patient reported that root canal treatment was performed on the left maxillary second molar by a general dentist due to dental caries five years ago. Intraoral examination of the left maxillary second molar revealed a defective class 2 composite restoration of the mesial side and secondary caries. The tooth was tender to percussion but not painful on palpation. The tooth exhibited grade 1 mobility and the periodontal probing depth was within the acceptable range. No abnormalities were detected on extraoral examination. Radiographic examination of the maxillary left second molar revealed caries on the mesial side, a missed distobuccal canal, apical radiolucency, extruded gutta-percha, and a separated file mid-length of the palatal root (Figure 1).

After a complete evaluation, according to the American Association of Endodontists in 2009, the diagnosis was previously treated therapy with symptomatic apical periodontitis. Treatment options were presented to the patient, including non-surgical endodontic retreatment with or without periapical surgery, or extraction with subsequent replacement of the missing tooth. The patient provided informed consent for non-surgical endodontic retreatment.

**Figure 2:**
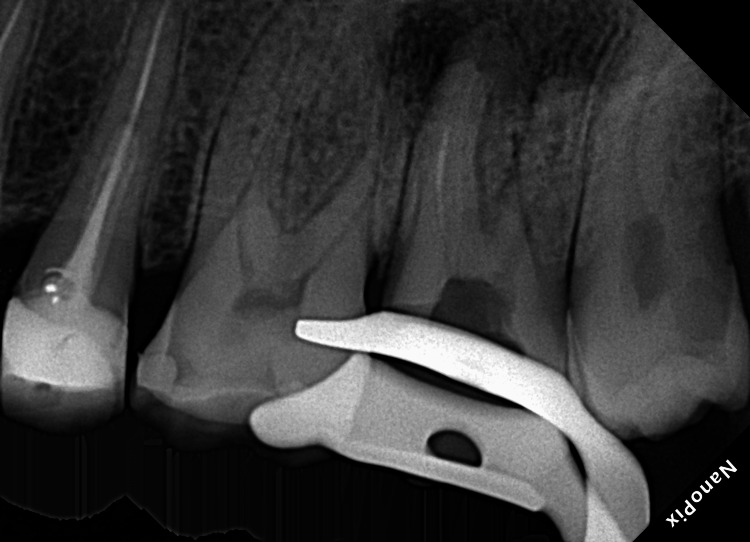
Intermediate radiograph after removed extruded gutta percha and separated file.

Following local anesthesia (2% lidocaine with 1:100,000 epinephrine infiltration), the involved tooth was isolated with a rubber dam. Treatment was performed under a dental operative microscope (Zeiss Extaro 300, Carl Zeiss Meditec AG, Oberkochen, Germany). Caries and the defective restoration were removed. Gates Glidden (GG) drills (sizes 2 and 3; Dentsply Maillefer, Ballaigues, Switzerland) were used to remove gutta-percha from the coronal portion of all canals. Subsequently, the palatal canal was retreated using two Hedstrom files (Dentsply, Maileffer, USA). A size 15 Hedstrom file was inserted along the gutta-percha to engage the extruded gutta-percha without using a solvent. Due to the size of the palatal canal and substandard previous obturation material, these allowed to insertion of the files around the gutta-percha and separated file then pulled these files together. Gutta perch in mesiobuccal canal was removed using the ProTaper Retreatment Kit (Dentsply Maillefer; Ballaigues, Switzerland), which is an innovative root therapy system consisting of three nickel-titanium (NiTi) instruments of varying lengths (16 mm, 18 mm, and 22 mm for D1, D2, and D3, respectively), diameters, and tapers (30/09, 25/08, 20/07) with chloroform. A radiograph was captured to verify the removal of gutta-percha and the separated file (Figure 2).

**Figure 3:**
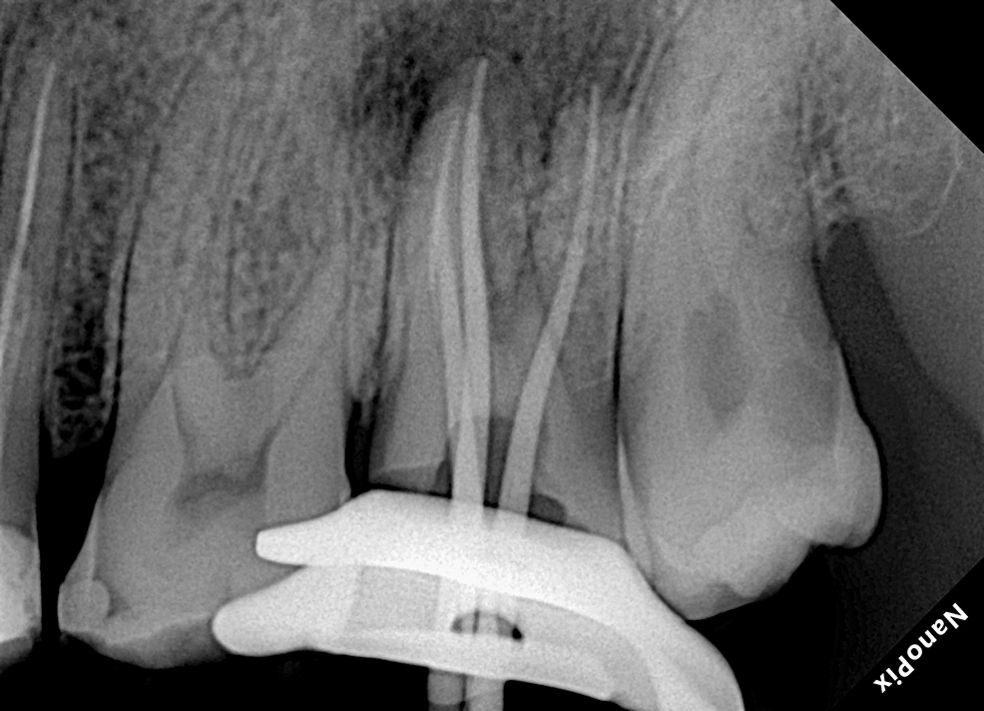
Mid-operative radiograph to show the fit of the gutta-percha cone

An electronic apex locator (Root ZX Mini, Morita, Osaka, Japan) was used to determine the working lengths. Cleaning and shaping procedures were performed using ProTaper Gold files (Dentsply-Maillefer, Switzerland), ensuring intermittent copious irrigation with 5.25% sodium hypochlorite in a side-vent needle (Figure 3).

**Figure 4:**
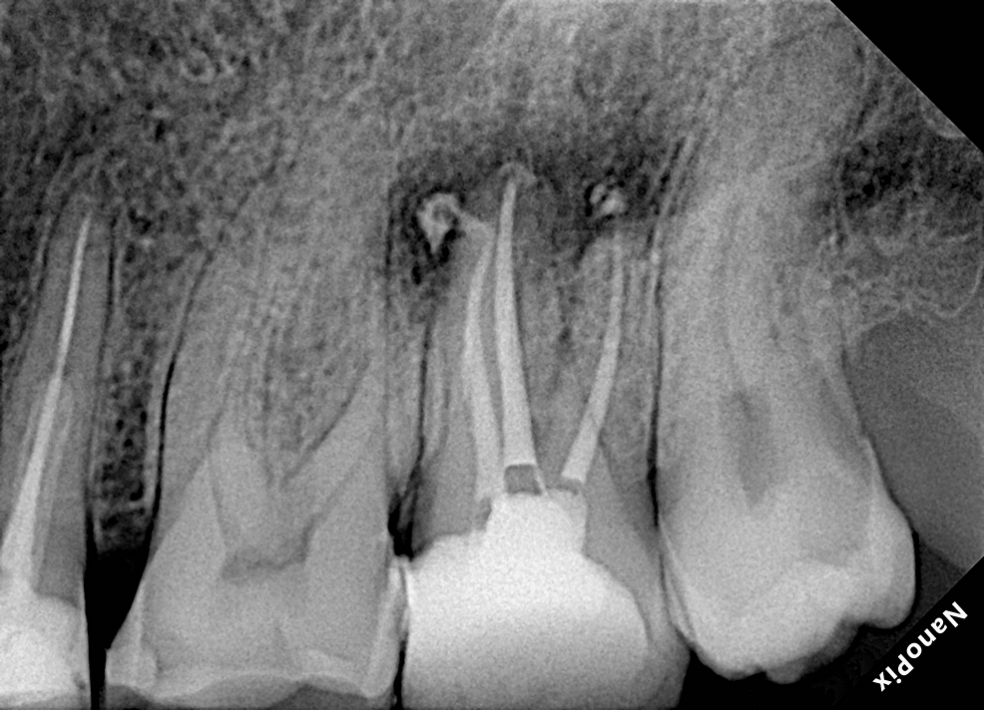
Postoperative periapical radiograph after the completion of root canal filling procedures

Sterile paper points were used to dry the canals, followed by the application of a bioceramic sealer (TotalFill BC sealer, FKG Dentaire, Switzerland) for obturation. The single-cone technique used F3 gutta-percha for the palatal canal and F2 for the mesiobuccal and distobuccal canals. There is a slight sealer puff (Figure 4).

**Figure 5:**
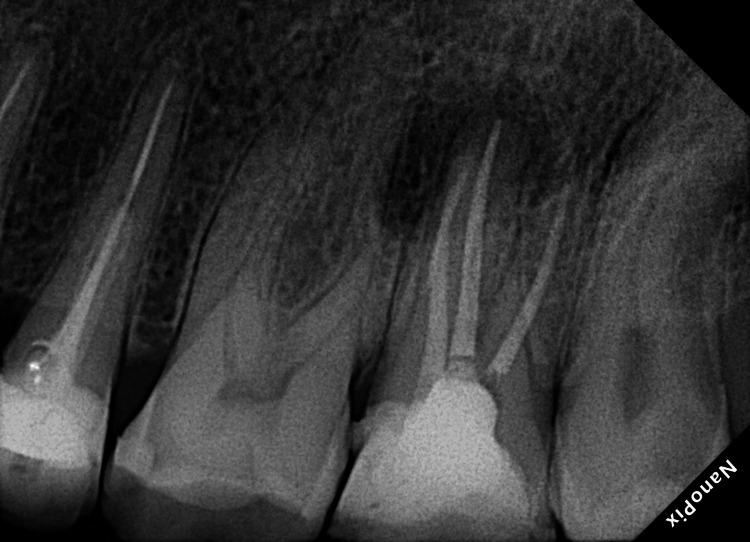
One-year follow-up radiograph showing healing of the periapical lesions

The patient was returned to the general dentist for final restoration of the treated tooth. At the 1-year follow-up, healing was evident at the apical region with sealer puff resorbed (Figure 5).

## Discussion

When root canal therapy is unsuccessful in treating initial periapical infection, tooth retreatment using conventional methods is recommended (particularly if the initial treatment is technically deficient). The successful application of antimicrobial agents to root canal dentin requires the removal of previous root-filling and other restorative materials. Procedural errors should be corrected. It is imperative to eliminate all materials to ensure the desired distribution of antimicrobial agents on all surfaces of the root canal dentin. According to a recent systematic review and meta-analysis of 33 cross-sectional studies conducted in multiple countries, the prevalence of apical periodontitis in 28,881 endodontically treated teeth was 36% [7].

Overextension of root canal filling materials occurs mainly because of a lack of material control [8,9]. The degree of material extrusion into the peri-radicular tissues depends on the techniques used for root canal preparation and obturation [10]. It may involve accidental overinstrumentation and incorrect canal working length measurements. Caution should be exercised when obturating a canal with open apices and larger minor foramen diameters, which are often observed in cases of radicular inflammatory resorption. An alternative method for handling overextended gutta-percha involves the use of a new Hedstrom file. By carefully inserting the file into the extruded apical fragment of the root filling and rotating it clockwise, it can be advanced slightly beyond the apical constriction by 0.5 mm to 1 mm. This helps to engage the overextended root filling. To complete this process and effectively remove the overextended material, the file should be gradually and firmly withdrawn without rotation [11]. The application of a solvent to soften the overextended apical fragment should be avoided, as it can compromise the ability of the Hedstrom file to firmly grasp the apical extrusion [12]. Of note, most root-filling removal techniques mentioned previously, such as heated pluggers, ultrasonics, rotary files, and solvents, are not safe choices for removing extruded portions of gutta-percha [13-15].

An operator's limited understanding of the tooth anatomy, complexities in canal configuration, or procedural errors (such as inadequate access cavity design) can contribute to a missed canal in an endodontically treated tooth. Missed canals are potential microbial reservoirs, and thus, the main cause of persistent apical periodontitis [16]. The endodontic literature provides clear evidence supporting the non-surgical endodontic retreatment of teeth with a significant number of missed canals following the initial endodontic treatment. Missed canals are identified in 42% of teeth that have been treated using non-surgical endodontic therapy [17].

The removal of separated instruments from root canals requires non-surgical orthograde procedures (mechanical or chemical) or surgery. Mechanical removal using specifically designed tools, such as braiding technique, extractors, wire loops, post-removal systems, ultrasonics, and laser irradiation, is common. The objective of the braiding technique is to free the fragment through filing, to engage it in the flutes of the file, and to retrieve it. The capture and removal of the fragment are facilitated by the braiding technique, with which two files are gently screwed into the canal alongside the fragment and then wound around each other until the fragment is tightly grasped and withdrawn. The successful removal of separated instruments depends on various factors, including the position of the instrument in relation to the canal curvature, the depth of the instrument within the canal, the type of separated instrument, fragment size, operator’s skills and qualifications. The rate of intracanal fractures of endodontic hand instruments varies from 0.25% to 6% [18]. Efficient disinfection and shaping are crucial for treating infected root canals, necessitating the removal of infected pulp tissues and any obstacles. The presence of a separated instrument presents a significant obstacle, with a potential risk for intracanal corrosion. Importantly, a separated instrument may restrict or occlude access, thus, preventing effective chemomechanical preparation of the canal beyond the separated instrument. Therefore, it is imperative to remove the separated instrument or bypass it to access the full length of the canal [19]. The clinician must inform the patient of the instrument separation and discuss the available treatment options, as well as the potential complications of both separated instrument retention and its attempted removal. The strength of this case report is the successful management of multiple endodontic mishaps in a single tooth. However, one of the limitations of this case report is that the remaining sealer was not removed completely, and the overhang composite restoration was due to the general dentist. Unfortunately, the patient did not come back to adjust the restoration.

## Conclusions

The accurate interpretation of periapical radiographs is paramount. Intermediate radiographs should be obtained if complications, such as a separated instrument, over-instrumentation, or missed canals are suspected by the clinician. Using the braiding technique with a Hedstrom file can help with removing extruded gutta-percha and separate files from a wide canal.
